# Sex as a Determinant of Age-Related Changes in the Brain

**DOI:** 10.3390/ijms25137122

**Published:** 2024-06-28

**Authors:** Dmitriy E. Burmistrov, Sergey V. Gudkov, Claudio Franceschi, Maria V. Vedunova

**Affiliations:** 1 Prokhorov General Physics Institute of the Russian Academy of Sciences, 38 Vavilova St., 119991 Moscow, Russia; dmitriiburmistroff@gmail.com; 2 Institute of Biology and Biomedicine, Lobachevsky State University of Nizhny Novgorod, 23 Gagarin Ave., 603022 Nizhny Novgorod, Russia

**Keywords:** brain aging, age-related brain changes, accelerated aging, gender differences, neurodegeneration, estrogenic support

## Abstract

The notion of notable anatomical, biochemical, and behavioral distinctions within male and female brains has been a contentious topic of interest within the scientific community over several decades. Advancements in neuroimaging and molecular biological techniques have increasingly elucidated common mechanisms characterizing brain aging while also revealing disparities between sexes in these processes. Variations in cognitive functions; susceptibility to and progression of neurodegenerative conditions, notably Alzheimer’s and Parkinson’s diseases; and notable disparities in life expectancy between sexes, underscore the significance of evaluating aging within the framework of gender differences. This comprehensive review surveys contemporary literature on the restructuring of brain structures and fundamental processes unfolding in the aging brain at cellular and molecular levels, with a focus on gender distinctions. Additionally, the review delves into age-related cognitive alterations, exploring factors influencing the acceleration or deceleration of aging, with particular attention to estrogen’s hormonal support of the central nervous system.

## 1. Introduction

Currently, numerous theories of aging exist, which can be broadly categorized into two groups: aging as a genetically programmed phenomenon and aging as a consequence of stochastic events not under genetic control, which entail cumulative damage to the organism over its lifespan [1,2]. Environmental factors are believed to influence at least 70% of human longevity [3]. Increasing evidence suggests that lifestyle choices, dietary habits, environmental influences, and exposure to toxins, including substance abuse, may have profound effects on lifespan and the occurrence of neurodegenerative diseases, although the precise molecular mechanisms underlying these effects remain largely unknown [4,5,6,7]. Consequently, healthy aging, characterized by the absence of disease development, may not be attributable to longevity genes, but rather to the absence of risk factors for neurodegenerative, oncological, and cardiovascular conditions [8]. Aging represents the principal etiological factor in the majority of neurodegenerative diseases, leading to cognitive decline and substantially impacting the quality of life in older individuals [9].

During the aging process, the central nervous system undergoes several irreversible changes affecting biochemical and molecular processes, leading to cellular-level alterations and morpho-anatomical rearrangements within the brain. Key structural changes associated with aging encompass tissue atrophy, functional activity redistribution across brain structures, neurotransmitter expression alterations, and the accumulation of cellular damage [10].

At the cellular level, aging is marked by oxidative stress development, reduced synaptic plasticity, diminished myelin synthesis rates, impaired calcium signaling, and metabolite accumulation. Age-related neurodegeneration correlates with disturbances in energy metabolism and astrocyte degradation, potentially diminishing both the quantity and quality of synaptic connections [11].

Well-documented cognitive changes during normal aging encompass deficits in new information processing and assimilation, memory, thinking, attention, information processing speed, language abilities, and spatial thinking [10,12,13]. Such cognitive decline can result in various challenges affecting daily functioning and overall well-being. Notably, significant heterogeneity exists in the rate of decline and overall cognitive function level among individuals of the same chronological age. While age-related neurodegeneration is intrinsic to the aging process, its accelerated progression marks a critical juncture, transitioning from normal bodily function to mild cognitive impairment and eventually to dementia [14].

A substantial body of evidence has accumulated regarding the presence of sex differences in the structure and progression of brain aging, contingent upon an individual’s sex. These disparities are attributed to the presence of a second X chromosome in females, as well as sex hormones such as estrogen and testosterone [15,16]. Despite the Y chromosome containing only 50–60 genes, the X chromosome harbors a considerable number of genes, many of which pertain to brain function. However, the precise role of X-linked genes in aging and the onset of neurodegenerative diseases remains inadequately understood [17]. Furthermore, approximately 10% of X chromosome genes evade inactivation during lyonization and exhibit varying expression levels across different bodily tissues [18]. Cumulatively, these factors underlie numerous sex differences in processes concerning information acquisition, perception, and retention [19]. Disparities in the prevalence of disorders, neurodegenerative diseases, and cognitive impairment based on sex have long intrigued researchers worldwide [20]. Over time, myriad molecular and cellular mechanisms have been elucidated, delineating the influence of sex hormones on these intricate processes. Notably, steroid hormones contribute to brain development and homeostasis maintenance. Estrogens, vital for optimal central nervous system function, modulate neurotransmission [21] and exert neuroprotective, neurotrophic [22,23], and anti-inflammatory effects [24].

Identifying sex-related structural differences in brain development and aging is the most important modern task, as it allows us to identify risk factors and differences in the course of central nervous system diseases in individuals of different sexes [25]. Thanks to the rapid development of non-invasive precision neuroimaging methods, including modern optical methods [26,27], it has become possible to obtain detailed structural and functional maps of the brain [28].

This review encompasses recent experimental findings elucidating sex differences in morphological and functional alterations observed in animal models and the human brain over the past several years. The cerebral cortex, hippocampus, and cerebellum, which are pivotal brain regions associated with cognitive functions, exhibit the most significant changes [29]. Moreover, the review scrutinizes factors contributing to accelerated and decelerated aging, with a particular focus on the pivotal role of sex hormones, particularly estrogens, in governing the processes of brain aging.

A comprehensive search of the literature was conducted using the scientific search systems PubMed, Google Scholar, and Web of Science. The review was primarily based on a critical analysis of scientific publications over the past two decades. The primary search terms employed in the literature review were “sex differences”, “morphological differences”, “structural differences”, “brain aging”, “neurodegenerative diseases”, etc. and their various combinations. In total, over 320 sources were analyzed. The search results were limited to those provided by the search services.

## 2. General Changes in the Brain Aging

### 2.1. Morphological Age-Related Brain Modifications

**Figure 1:**
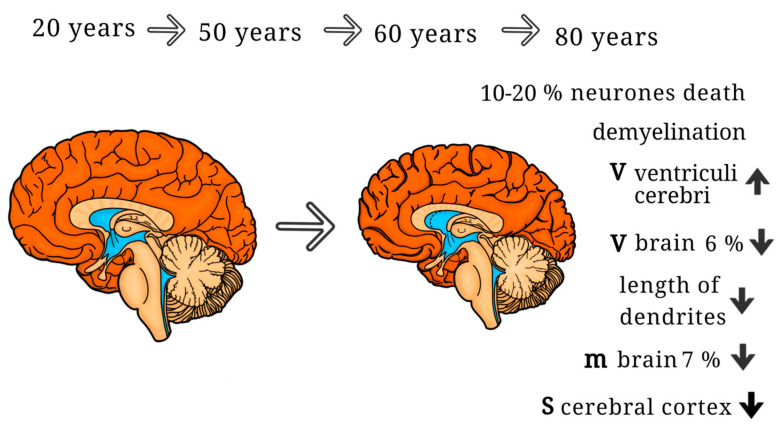
Key structural changes in the aging brain; V—volume; S—square; m—mass.

The aging process of the brain is characterized by a reduction in its overall mass and volume over time. Between the ages of 60 to 75 years, the human brain experiences a decrease in mass of 6%, although this decline occurs disparately across different brain regions [30]. Primarily, the temporal and frontal lobes exhibit a notable reduction in volume with age [31]. This loss of volume is often accompanied by an enlargement of the ventricles and other cerebrospinal fluid (CSF) spaces [32,33] (Figure 1). Early studies conducted by Brody [34] and Chugani et al. [35] reported a loss of up to 25% of Purkinje cells in the cerebellum and up to 18% of nuclei in the thalamus. Gray matter volume begins to exhibit a slight decline after the age of 20 and continues to decrease linearly with advancing age [36]. Furthermore, brain aging is characterized by the deterioration of white matter, involving the loss of myelinated fibers and disruptions in myelin sheath formation [37,38,39]. White matter degeneration is particularly prominent in the frontal brain regions and the corpus callosum [40,41,42]. Complete demyelination of nerve fibers can impair conductivity and reduce connectivity between brain regions, consequently slowing down information transmission [43,44].

As individuals age, the cerebral cortex undergoes a reduction of approximately 4%, with the most notable changes occurring in the frontal lobe, where reductions of 12–15% have been observed [45]. Gray matter loss in the cortex is generally more pronounced compared to subcortical structures, except for the hippocampus. This loss of gray matter is associated with decreased arborization complexity and dendritic shortening, a reduction in the number of dendritic spines, decreased synaptic density and transmission, ultimately leading to cognitive decline [33,46]. The atrophy of nerve cells in the frontal regions of the brain contributes to difficulties in problem-solving and significantly diminishes overall quality of life for elderly individuals [10]. Consequently, the principal morphological and functional changes in the cerebral cortex and their correlation with age-related declines in motor and cognitive functions have been well-established. Other brain regions, such as the midbrain, hippocampus, and cerebellum, are also implicated in age-related degenerative processes and warrant further investigation.

Furthermore, structural alterations in the brain’s membranes are evident with aging. The dura mater thickens and becomes sclerotic, eventually fusing with the skull bones. Similarly, the pia mater undergoes noticeable thickening, with convolutions becoming thinner and grooves widening and deepening, while the arachnoid membrane becomes hyperplastic and sclerotic [47]. Age-related decreases in angiogenesis lead to reduced vascular density [48] and increased stiffness of the vascular walls in the brain [49]. Consequently, there is a decline in blood flow and metabolism in brain cells, accompanied by a deceleration of cognitive processes [50,51].

#### Sex Differences in Brain Structure and Changes Associated with Aging

On average, men have a total brain volume approximately 8–15% larger (equating to 110–115 g) than that of women [52]. Research indicates that brain weight decreases in men by 2.7 g per year and in women by 2.2 g [53]. Utilizing fMRI analysis, Armstrong et al. investigated the brains of 295 men and 328 women, revealing that sex differences in volume loss affect the entire brain. The annual rate of decline in brain volume is more pronounced in men across most areas, with the exception of white matter loss in the temporal and occipital regions. Additionally, males demonstrate greater enlargement of the cerebral ventricles [54]. Further studies have indicated that reduced cortical volume [55] and deficits in glucose metabolism during aging are more prevalent in men [56]. In a morphometric investigation utilizing MRI data from a cohort of 411 individuals, Chen et al. identified regional sexual dimorphism in gray matter distribution among middle-aged subjects (44–48 years). Specifically, males exhibited increases in gray matter in regions such as the midbrain, left inferior temporal gyrus, right occipital lingual gyrus, right middle temporal gyrus, and both cerebellar hemispheres [57]. Conversely, women demonstrated greater cortical gray matter volume as well as a higher gray-to-white-matter ratio in the brain [58]. Additionally, numerous studies have reported that men typically have a larger amygdala and thalamus compared to women, while women tend to have a larger hippocampus, regardless of age [59,60,61,62]. Wang et al. conducted a study utilizing MRI data to analyze the volumes of subcortical structures, including the right putamen, right pallidum, bilateral thalamus, hippocampus, and amygdala, across individuals of various sexes and ages; they revealed that the volume of the right putamen, right pallidum, and right thalamus decreased more rapidly in males than in females, and the volume of the left thalamus, bilateral hippocampus, and amygdala in males followed a quadratic model, while those in females followed a linear decline model [63]. Several prior studies have also investigated sex differences in brain structure and yielded comparable findings [57,64,65].

Given the considerable variability in the rate of change across brain regions with aging, we will provide an overview of key morphometric and functional changes across regions, along with a more detailed examination of sex differences in brain aging in the subsequent sections (Section 2 and Section 3).

### 2.2. Cellular Changes in the Brain during Aging

**Figure 2:**
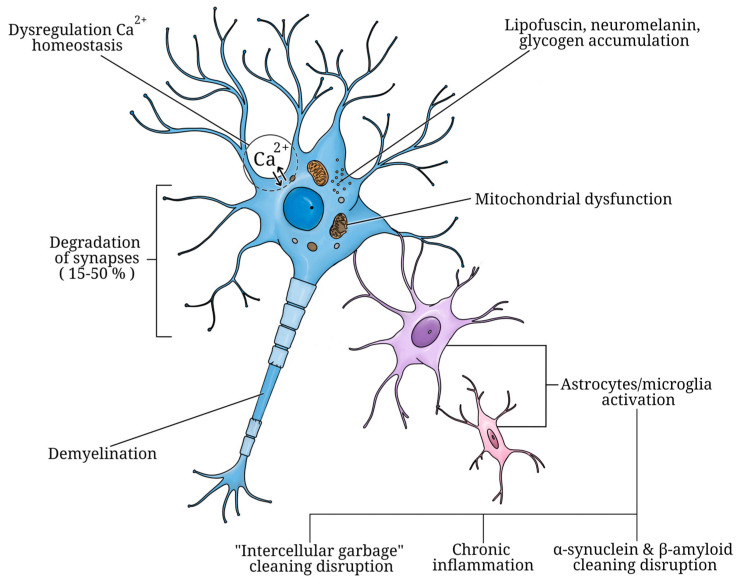
Brain cells transformations associated with aging.

At the cellular and molecular levels, normal aging is accompanied by intraneuronal accumulation of lipofuscin and neuromelanin pigment by nerve cells [66], disorders of neuronal morphology manifested in a decrease in the density of dendrites [67]. Glycogen inclusions, degenerated mitochondria, and accumulations of filaments in axons are observed. Neurons and their processes decrease in size; lipofuscin and fat vacuoles accumulate in them [66]. Not only signs of damage and dystrophy of neurons (homogenization of the cytoplasm, displacement and pycnosis of nuclei, cytolysis, tigrolysis) of various severity but also signs of hypertrophy of intracellular structures are revealed; they indicate adaptive processes in conditions of age-related neuronal degeneration [10,68] (Figure 2). Due to neuronal death, one of the typical morphological signs of the aging brain—rarefaction of cells—arises [69,70]. Voids in areas of complete disappearance of neurons contain granular basophilic material and vacuoles. In the context of age-related changes in neuronal count, divergent findings have emerged. On one hand, there is evidence suggesting that substantial neuronal loss transpires within the central nervous system as a consequence of normal aging; a process often compensated by glial cell replacement [71,72]. Other reports suggest that the glia/neurons ratio remains stable across aging in the CNS, while observing heightened activation of glial cells alongside extensive neuronal connection and network losses [73,74,75,76]. Typically, neuronal loss is limited to specific areas of the central nervous system and does not exceed 10% [77,78].

Furthermore, aging prompts the accumulation of damaged and aggregated proteins within neurons, alongside impaired mitochondria, due to heightened oxidative stress, which arises from escalated reactive oxygen species (ROS) production and/or diminished antioxidant system activity [79,80,81].

The density of synapses decreases with aging [82]. The total number of synapses decreases at a rate of 15 to 50%, depending on the CNS department [31]. However, the loss of synapses does not occur in all parts of the CNS to the same extent. For example, in the frontal lobe [83], a decrease in the number of synapses with age has been reliably proven, while in the temporal lobe, age-related changes are not observed. Changes in synaptic function are evident not only in the cortex but also in subcortical structures. For instance, age-related deficits in spatial memory have been linked to diminished specificity, efficacy, and plasticity of synaptic transmission within the hippocampus [84]. Additionally, the capacity to form new synapses diminishes with age [85]. Reduced synaptic plasticity in older adults may contribute to memory impairment and decline in motor function [86]. Neurons experience deafferentation, leading to compromised responsiveness to environmental cues, neural, and hormonal stimuli [87].

Moderate biochemcal and metabolic changes caused by the changes in the expression of synaptic transmission mediators and their receptors in various parts of the brain are noted [88]. With aging, the state of the mediator systems of the brain changes significantly. One of the most distinctive aging phenomena is the degeneration of the brain’s dopaminergic system, which is directly related to the development of diseases such as Parkinson’s disease (PD) in old age. Disturbances in the activity of the cholinergic mediator system of the brain play one of the main roles in disorders of memory, perception, and other cognitive processes that occur in Alzheimer’s disease (AD). In addition to the above disorders, aging is also characterized by impaired cellular waste removal mechanisms [89], dysregulation of Ca^2+^ homeostasis [90,91], and mitochondrial neuronal dysfunction [92]. These alterations occur in normal aging and are exacerbated in vulnerable neuronal populations in neurodegenerative diseases [78]. Astrocytes and microglia undergo activation [73,93]. Age-related changes in glial cells can cause persistent chronic inflammation, making the brain more susceptible to neurodegenerative processes [94,95]. The aging process and the emergence of age-associated neurodegenerative conditions coincide with disturbances in lysosomal autophagy processes and the ubiquitin-proteasome clearance system executed by glial cells. Consequently, this leads to the accumulation of so-called “cell garbage” within brain tissue, characterized by the presence of damaged molecules encompassing lipids, proteins, and DNA/RNA [10,96]. Changes in the expression levels of inflammatory mediators by microglial cells contribute to the onset of neurodegenerative pathologies [97]. “Young” microglia exhibit superior efficiency in phagocytizing Aβ [98] and α-synuclein [99] compared to their aged counterparts. Bonham et al. [100] utilized a microglia-specific marker (TMEM119) and a cell type expression profiling tool (CellMapper) to reveal that normal aging induces increased expression of 30 previously unidentified microglia-specific genes in several regions of the healthy human brain as well as those affected by AD.

Myelin fibers become thinner with aging [101]. Violation of myelination negatively affects the efficiency of neural processes in the brain responsible for the production of this important substance. Over time, the expression by oligodendrocytes of one of the main genes responsible for the production of myelin *GPR17* decreases, which also reduces the activity of cells that provide the myelination of fibers [102,103].

During brain aging, amyloid-beta (Aβ) deposits are also revealed [104]. These deposits are situated adjacent to the vessels of the microvasculature of the cortex and constitute accumulations of argyrophilic structureless material containing amyloid, surrounded by tangles of thickened axons and macro- and microglial cells. Aβ may accumulate in the brains of individuals classified as cognitively normal, yet this may indicate a heightened risk of developing cognitive impairment over time [12].

## 3. Key Sex Differences in Brain Aging

A number of studies propose that alongside sex-related structural differences in the brain, sex disparities exist in the dynamics of brain aging, the progression of age-related neurodegenerative diseases, and the onset of dementia. However, the intricacy of this relationship remains multifaceted [105,106,107,108]. A systematic search by Wrigglesworth et al. assessed 52 studies exploring brain aging in clinical and community-dwelling adults (mean age between 21 to 78 years, ~37% were female) [107]. Employing machine learning age prediction using neuroimaging (MRI) data, Cole et al. [105] found that the brain age predicted from neuroimaging data in older females was, on average, younger than their chronological age, while the converse was observed among males. In a separate study, Gole et al. examined positron emission tomography scans of the brains of 205 adults (aged 20–82 years) and observed that, in terms of metabolic parameters (such as measures of regional glucose uptake, oxygen uptake, and cerebral blood flow), women’s brains, on average, appeared several years younger than those of their male counterparts [109]. Smith et al., based on MRI data of 108 females and 76 males, estimated non-linear brain age and found that, according to a comprehensive analysis, females had a mean brain age delta that was 0.7 years higher than in males [110].

### 3.1. Sex-Depending Age-Related Cognitive Differences

**Figure 3:**
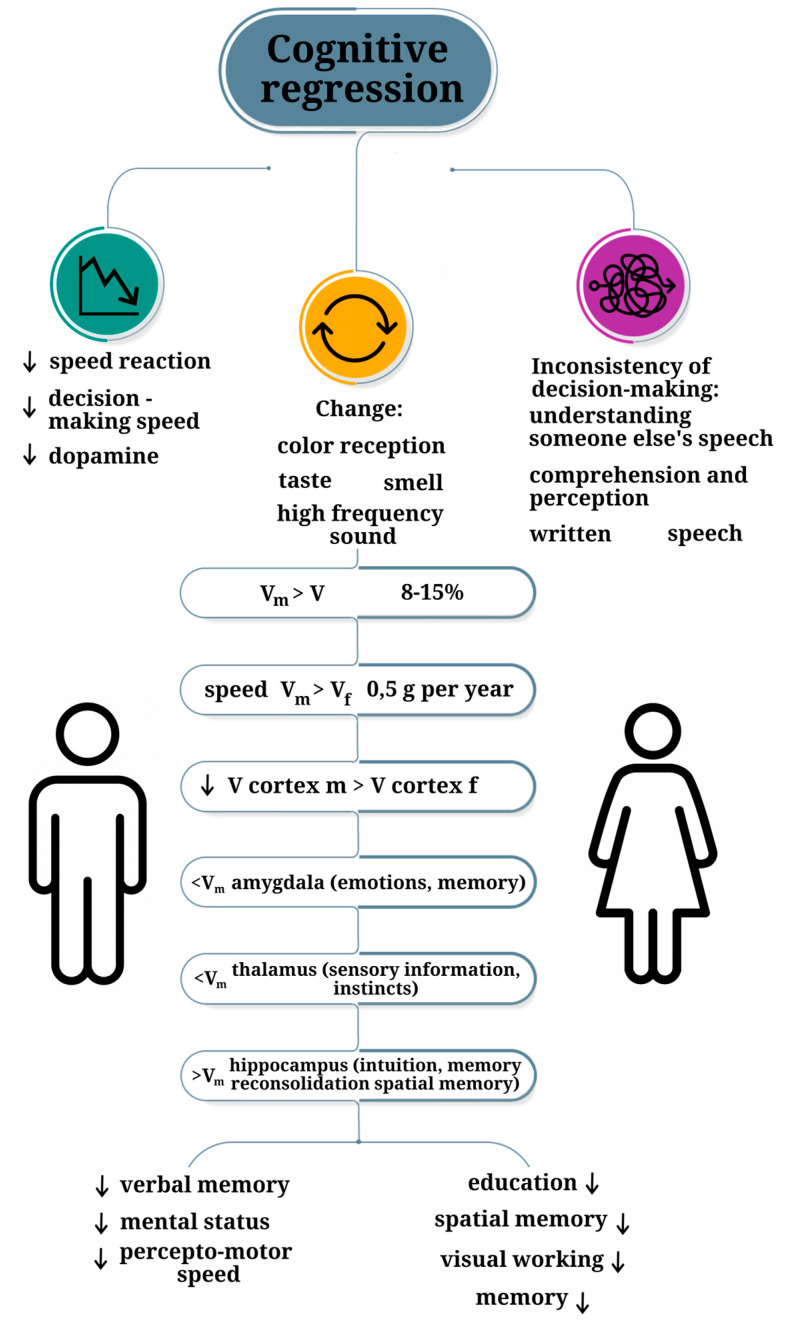
Key age-related brain morph-functional and cognitive transformations, depending on sex; V_m_—volume of male structures; V_f_—volume of female structures; m—male; f—female.

Age-related cognitive changes encompass alterations in the speed and accuracy of decision-making, disruptions in the perception of external stimuli (such as color, taste, smell, and sound), memory decline, and reduced speech rate (Figure 3). Investigations into decision-making behavior among older adults have indicated a tendency to employ simpler heuristic strategies [111]. Furthermore, recent studies have suggested that the gradual decline in reaction times, noticeable from the age of 20 onwards, is linked to heightened caution during decision-making processes and a decelerated pace of decision-making, rather than alterations in thought speed [112]. Diminished efficiency in decision-making processes is attributed to cognitive limitations in information processing that typify aging [113]. Moreover, older individuals have been observed to display lesser consistency in their choices [114]. From a biological perspective, these effects can be rationalized by the demise of neocortical neurons, with pronounced atrophic changes being evident in the prefrontal cortex. Additionally, there is a reduction in overall synapse count and a decline in the synthesis of numerous neurotransmitters, which serve as primary information carriers between neurons [115]. Recent attention has also been drawn to age-related shifts in color perception [116,117]. Age-related effects are most prominently observed in shade discrimination, where sensitivity in the short-wave range of the spectrum diminishes more significantly with age compared to the medium- and long-wave ranges [118].

Explorations into the connection between age-associated alterations in cognitive abilities and sex hold particular significance [11]. It is noteworthy that numerous studies have demonstrated the relationship of age-related cognitive impairment with gender, observed both in model animals and in humans (Figure 3). For instance, a pronounced decline in overall cognitive function during advanced age has been evidenced in females, surpassing that observed in males [119]. In a study by Benice et al., old mice (18–20 months old), irrespective of sex, exhibited impaired spatial water maze performance during training compared to young or middle-aged mice. Notably, only old females failed to demonstrate robust spatial bias during probe trials. Moreover, males did not show age differences in passive avoidance scores, unlike females [120].

In humans, males exhibit superior performance in spatial memory and spatial orientation tasks [121]. This distinction persists even in later life [122]. Conversely, females consistently display superior verbal memory capabilities compared to males throughout their lifespan, implying the independence of this process from circulating sex hormones [123,124,125]. Importantly, these sex differences are based on averages and do not apply to all individuals [126]. Beyond these differences, there are many cognitive functions that are not significantly different between the sexes [127]. Additionally, a study conducted by Pauls et al. involving 366 females and 330 males aged 16 to 69, revealed that females exhibited superior performance on verbal memory tasks. Males across all age cohorts exhibit enhanced performance on visual episodic and visual working memory tasks [128,129,130]. A study involving 1065 to 2127 healthy participants (with a mean baseline age ranging from 64.1 to 69.7 years, according to the BLSA dataset) revealed steeper declines in mental status, perceptual speed, and integration among male participants. In contrast, no discernible indicators of sharper decline were identified in women. These findings underscore the heightened resilience to age-related cognitive decline observed in older women relative to men [131]. Importantly, cognitive decline associated with AD presents with differential patterns in females and males. Specifically, females tend to sustain their reserves of verbal memory for a more extended period compared to males [132,133].

The fundamental biological mechanism underlying the variance in cognitive functions among individuals of different sexes is widely attributed to the regulatory influence of sex hormones [122,134,135,136]. In several studies involving the exogenous administration of sex hormones, alterations in cognitive functions have been subsequently observed. For instance, in females of reproductive age, a solitary administration of testosterone at concentrations akin to those observed in males has been shown to enhance visuospatial abilities [137,138]. It is widely accepted that the impact of sex hormones on cognitive function commences as early as the prenatal stage of human development, a period during which the brain’s development diverges in response to androgen production [126]. In males, there occurs a surge in fetal testosterone between the 8th and 24th weeks of gestation, followed by a more subdued increase in testosterone around 3 to 4 months post-birth [139]. During this “critical window”, the male fetus generates over 2.5 times the quantity of testosterone compared to the female fetus [140], signifying this period as pivotal for hormonal influence on brain development, subsequently laying the groundwork for cognitive functionalities [139].

### 3.2. Sex as Age-Related Neurodegeneration Risk Factor

It is known that women are notably more vulnerable to AD and other forms of dementia than men, while PD is more prevalent in men, occurring twice as often [141,142]. Despite this distinctive distribution of PD between the sexes, this discrepancy remains insufficiently explored, partly due to women’s higher mortality and the swifter progression of the condition [143]. For both men and women, age and the associated decline in sex steroid hormones are considered pivotal factors influencing the risk of developing neurodegenerative diseases, including AD [144]. Indeed, several animal model experiments have unveiled a connection between sex and AD pathogenesis. Notably, the reduction in sex steroid production in female rodents via ovariectomy has been linked to a pathogenesis akin to AD, characterized by an elevation in brain Aβ levels [145]. Similarly, female transgenic APPxPS1 mice (expressing the amyloid precursor protein and presenilin 1) displayed more prominent cognitive impairments and deficits in hippocampal neurogenesis compared to their male counterparts [146].

The apolipoprotein E ε4 (ApoE ε4) genotype represents the most widely recognized genetic risk factor for late-onset AD, correlating with an augmented deposition of Aβ, a pathological hallmark of AD manifested in amyloid plaques [147]. Female carriers of the ApoE ε4 allele demonstrate a fourfold higher susceptibility compared to male carriers between the ages of 65 and 75 years [148,149]. Emerging data suggest that the APOE ε4 genotype might interact with the influence of exogenous estrogen exposure [150,151,152], impacting dementia and the risk of AD [153,154]. Moreover, sex plays a significant role in the pathophysiology of AD, particularly in the interaction between Aβ and phosphorylated tau. It was found that females, especially those who are carriers of the APOE ε4, demonstrate an increased susceptibility to tau accumulation in the presence of β-amyloid compared to males [155]. Additionally, Buckley et al. observed a tendency toward heightened tau accumulation in the cerebrospinal fluid of amyloid-positive female carriers of the APOE ε4 without evident cognitive impairment, a trend not replicated in males [156]. Thus, the ε4 allele of the apolipoprotein E4 gene (*APOE ε4*) amplifies the risk of AD more profoundly in females than in males [148].

Notwithstanding the well-established sex differences in the incidence of neurodegenerative disorders and dementia prevalence, the precise mechanisms underpinning these differences remain a topic of ongoing debate. Presently, several primary hypotheses have been advanced to elucidate the sex differences that contribute to accelerated aging and the emergence of age-associated neurodegenerative conditions, including AD. Undoubtedly, sex steroid hormones stand as pivotal risk factors for AD, as they constitute crucial determinants of the sex-based differentiation in brain structure and cognitive function [157]. Sex hormone receptors have a specific impact on nuclear transcription factors, leading to the modulation of transcriptional activity upon binding [158]. In addition to age-related decline in sex hormone concentrations in both sexes (such as estrogens, progesterone, testosterone), there are other fundamental factors that are also related to sex and cause both accelerated aging and the risk of developing age-related neurodegenerations. These factors encompass diverse genetic risks (prominently ApoE), comorbid conditions (including diabetes, obesity), depressive disorders, and cardiovascular diseases [122,159,160,161,162,163] (see Section 5 “Factors of accelerated aging”).

## 4. Age-Related Changes in Certain Parts of the Brain

### 4.1. Cerebral Cortex

**Figure 4:**
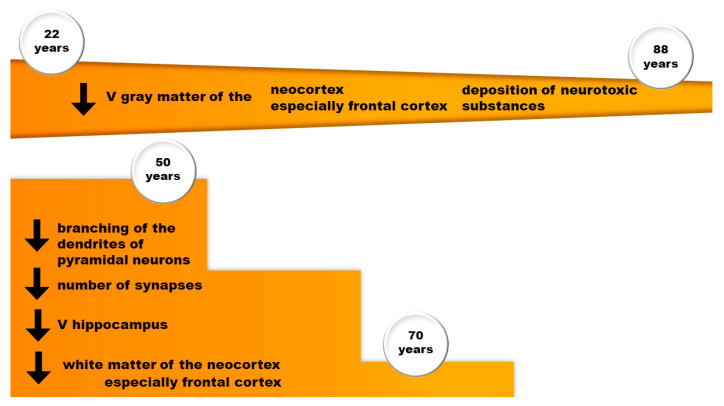
Dynamics of age-related changes in the brain; V—volume.

Brain aging (in the age range of 22–88 years) causes a decrease in the linear and quadratic sizes of key brain structures (Figure 4). At the same time, the sizes of the gray matter of the neocortex and frontal cortex decrease linearly, while the sizes of the hippocampus and the white matter of the neocortex and frontal cortex decrease non-linearly, with a sharp decrease in size at age over 70 years [164]. It is noteworthy that no such age-related changes were found for chimpanzees. The authors of the work attribute this phenomenon to an increase in life expectancy in humans compared to other primates [164]. Post-mortem studies of the human brain demonstrated degenerative changes in the microstructure of the neocortex with age. In particular, after the age of 50, there was a violation of the branching of the dendrites of pyramidal neurons and a decrease in the number of synapses in the associative areas of the neocortex and hippocampus [165,166,167]. Neuronal perikarya in the cerebral cortex also decrease in volume during human adulthood [168]. However, brain aging in healthy individuals is not accompanied by a significant loss of neurons in the human cerebral cortex [169]. Lu [170] used the structural magnetic resonance imaging (sMRI) scan data to perform multiscale measurements of cortical complexity, including thickness, surface area, gray matter volume, density, gyrification index, and fractal dimension in different age groups of people (young, middle-aged, young-old, and old-old).

In older adults, studies have identified a decrease not only in gray matter volume and brain surface area but also in the ratio of the total internal surface area to the outer surface area, which smoothly encompasses the cerebral cortex [170]. It is noteworthy to mention several longitudinal studies that have documented changes in morphometric parameters of the cerebral cortex with age. For example, Lemaitre et al. reported a decrease in the total surface area of the brain by 3.68 cm^2^ per year and the average thickness of the cortex by 0.004 mm per year [171]. Storsve et al. [172] also reported an average annual decrease in the cortical area of 0.19% per year, an average loss of cortical volume of 0.51% per year, and an average decrease in cortical thickness of 0.35 mm per year in a study group of 297 healthy people aged 23–87 years old.

Structural neuroimaging studies showed that the aging process proceeds differently in different areas of the cerebral cortex, with the frontal areas being particularly prone to atrophy [173]. In contrast to other regions of the central nervous system, the cerebral cortex exhibits a remarkable resilience to the processes associated with normal aging. While there is evidence of a gradual loss of synapses throughout the cortex with advancing age, the majority of neurons within this region appear to maintain their functionality throughout the lifespan [174].

Thinning of the cortex was greatest in the heteromodal association cortex and regions of high postnatal surface area expansion. In contrast, thinning in old age was insignificant in primary sensory cortices and regions of low postnatal surface area expansion. Degradation processes begin even later in the motor cortex [173]. The activity of the frontal lobes of the cortex decreases significantly with age. The decrease in cortical activity with age is most pronounced in the frontotemporal areas associated with attention, inhibition, and memory [65]. Over time, sharp peaks of activity during wakefulness disappear in individuals. Notably, speech, the functional centers of which are located in the frontal lobe of the cerebral cortex, is relatively well preserved with aging. Older people aged 60–70 use more diverse grammatical forms in their speech than younger people. The fluency of speech in the elderly does not differ from that of younger people. However, there are changes in the processes of understanding someone else’s speech due to sensory deficits and a slowdown in the speed of information processing. In relation to written speech, certain changes are also observed with age. Comprehension and perception slow down; it becomes more difficult for older people to grasp the meaning of what they read. This is probably due to the gradual deterioration in the functioning of the brain regulators of sleep and wakefulness [175]. In addition, the elderly has a worse tolerance to sleep deprivation [176,177]. With age, cortical thinning can be caused by the deposition of neurotoxic substances, and white matter degeneration can be caused by vascular abnormalities. These changes, occurring in the brain, jointly disrupt sleep spindles and slow-wave sleep, resulting in sleep disturbances. Age-related dysregulation of neurotransmitters (including galanin, orexin, serotonin, and adenosine) directly disrupts the sleep modulation system [178]. Lifelong bilingualism is associated with delayed diagnosis of dementia, suggesting that bilingual experience is relevant to brain health during aging [179]. Cabeza et al. [180] found that the reduction in hemispheric asymmetry observed in older people is most pronounced in the prefrontal cortex. It is still unclear whether the bilateral alignment is a reflection of compensatory activation of one of the hemispheres or whether it is the result of pathological changes.

When assessing post-mortem human brain samples from patients with AD, it was found that over 95% of aging neurons were excitatory neurons and had neurofibrillary tangle (NFT) pathology. The authors predicted that the cyclin-dependent kinase inhibitor 2D (CDKN2D/p19) contributes most significantly to the development of brain aging. The population of neurons expressing p19 had 1.8 times larger nuclei and significantly more cells with lipofuscin than p19-negative neurons. These typical aging phenotypes were enhanced in the presence of NFT. Thus, NFT neurons expressing CDKN2D/p19 represent a unique cell population in human AD with an aging-like phenotype [181]. The entorhinal cortex, which serves as a relay center between the hippocampus and association areas, undergoes early volume reduction in Alzheimer’s dementia but not normal aging [12].

### 4.2. Midbrain

**Figure 5:**
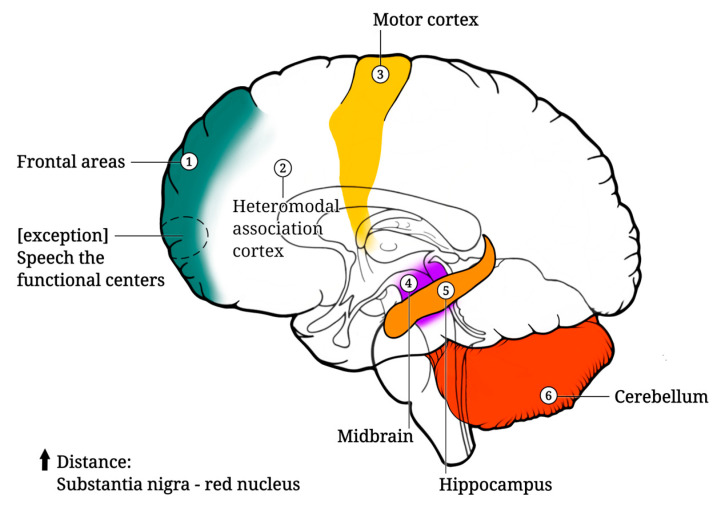
The most vulnerable parts of the brain during aging.

Based on MRI data, Doraiswamy et al. [182] observed a negative correlation between age and estimated midbrain volume, anteroposterior diameter through the substantia nigra, and interpeduncular distance. Interestingly, linear measurements for the right and left sides were nearly identical. Sohmiya et al. [183] utilized T2-weighted MRI to measure midbrain structures and found an overall age-related decrease in structural volume. They also reported a positive correlation between aging and the maximum distance of the substantia nigra (Figure 5). Notably, these midbrain regions exhibited significant left–right differences, including the region of the red nucleus, which may play a role in hemispheric dominance [183].

A number of studies emphasized functional age-related changes in midbrain structures that cause a decrease in dopamine receptors and transporters and the presence of a close relationship between dopamine synthesis in the midbrain and reward-related activity of the prefrontal cortex during normal aging [184,185,186,187]. A recent paper by Russo et al. [188] suggested that increased secretion of pro-inflammatory factors by senescent midbrain cells triggers neuroinflammation, leading to immune-cell-mediated killing of midbrain dopaminergic neurons in PD.

### 4.3. Hippocampus

Another brain structure of interest in the context of aging is the hippocampus, a region with certain functional and structural plasticity that plays an important role in learning, memory consolidation, affective behavior, and mood regulation. Changes observed in the aging hippocampus are thought to include increased oxidative stress and neuroinflammation, altered intracellular signaling and gene expression, and decreased neurogenesis and synaptic plasticity [189]. It is known that hippocampal neurons are especially susceptible to damaging external factors; their death is noted in the situations of emotional stress and under the influence of hypoxia. The deterioration of memory and general cognitive abilities with aging is largely caused by these features [190].

In the hippocampus, neuronal death may be partially compensated by neurogenesis [191,192]. The dentate gyrus of the hippocampus exhibits continuous neurogenesis throughout adulthood, contributing to learning and memory processes [193]. However, with age, neurogenesis is significantly impaired. In recent years, emerging evidence has shown that neurogenesis can restore a younger state during aging. Some evidence suggests that it is possible to influence longevity and aging through genetic manipulation [194]. Gene therapy aimed at genes implicated in the aging process represents a promising field of research with considerable potential. Notably, genes like *TERT* and *APOE* have advanced to clinical experimentation stages [195]. One of the genes of interest in this regard is the *Klotho* gene. The Klotho protein is involved in the regulation of phosphate and calcium metabolism, protects cells from oxidative stress and apoptosis [196], and suppresses intracellular insulin and insulin-like growth factor-I signaling pathways [197,198]. It has been shown that trauma at different stages of development can influence the nature of memory deficits and hippocampal atrophy [199].

Several studies have underscored sex differences not only in the structural and morphological composition of the hippocampus but also in its functional activity. These variations encompass the dependence of the plasticity of the hippocampus on the hormonal background [52,200]. Circulating hormones affect three forms of hippocampal structural plasticity: (1) repeated stress causes remodeling of dendrites in the CA3 region; (2) various modalities of stress suppress neurogenesis of dentate gyrus granule neurons; (3) ovarian steroids regulate synapse formation during the estrous cycle of female rats. All three forms of structural remodeling of the hippocampus are mediated by hormones working in concert with excitatory amino acids (EAA) and NMDA receptors. EAA and NMDA receptors are also involved in neuronal death that is caused in pyramidal neurons by seizures, ischemia, and severe and prolonged psychosocial stress. The aging brain appears to be more vulnerable to such effects, although there are significant individual differences in vulnerability that can be developmentally determined. For example, it has been shown that the brain’s resistance to stress is associated with estrogen [201]. It has been observed that exposure to stress significantly diminishes the proliferation activity of the hippocampus in male rats but not in females [202,203]. In addition, changes in the proliferation activity of the hippocampus were recorded in female animals depending on the hormonal background [204,205,206]. Chow et al. observed a difference in the activity of neurogenesis after learning the spatial task in male and female rats. Spatial learning regulated neurogenesis in the hippocampus differently in females and males, albeit the activity of new neurons in response to the restoration of spatial memory was similar [207].

### 4.4. Cerebellum

The cerebellum is a part of the brain located at the back of the head and is responsible for the coordination of movements, regulation of balance, and muscle tone [208,209,210].

There is sufficient data on functional changes in the cerebral cortex and the relationship with age-related decline in motor and cognitive functions; however, the potential involvement of such a brain region as the cerebellum in these processes is less clear [211]. It is established that the cerebellum undergoes significant morphological transformations with age, characterized by a reduction in its overall volume [212,213], which is caused by a decrease in both gray and white matter within the cerebellum [214]. Several studies have reported a reduction in the volume of the cerebellar vermis in association with aging [212,215]. In parallel, early studies conducted by Raz et al. [216,217] revealed a noteworthy age-related reduction in the volume of the cerebellar hemispheres. A morphometric MRI study by Tiemeier et al. [218] shows that total cerebellar volume in individuals aged 5 to 24 years reaches a peak at about 12 years in females and 16 years in males. In turn, Wu et al. determined that cerebellar volume increases logarithmically, with an initial rapid increase during the first 2 years of child development. This increase occurs earlier in girls, especially in the case of total cerebellar volume and unmyelinated brain tissue [219]. A study of MRI data obtained from 190 healthy individuals spanning an age range of 18 to 81 years, conducted by Raz et al., revealed sex-related age-dependent morphometric disparities within the cerebellum. Notably, even after accounting for height differences, the volume of cerebellar hemispheres, vermis, and ventral pons was larger in males [217,220]. Correspondingly, Sussman et al. observed contrasting results in terms of the relative volumes of the cerebellum based on sex: white matter volume exhibited a significant increase in females, whereas cerebellar lobule volume was greater in males. A recent study by Stalter et al. [221] investigated morphometric changes in the cerebellum of the aging brain based on data from 3 T-MRI scans; there were study groups of 24 older individuals (64.42 ± 4.8 years) and 25 young individuals (24.6 ± 2.14). An age-related decrease in the volume of gray matter in the right regions of the cerebellum, functionally associated with non-motor networks and areas of cognitive tasks of the cerebellum, was revealed. In the study of Andersen et al., a histological analysis of the human brain of various ages (19–84 years) was performed. A decrease in the global volume white matter of 26% was found; at the same time, the average volume of the body of Purkinje cells decreased by 33% without a decrease in the volume of nuclei. There was also a trend towards a decrease in total cerebellar volume by 16% without concomitant loss of neurons [222]. Koppelmans et al. investigated the association of cerebellar gray and white matter volumes with motor activity in the elderly [223]. The gray and white matter of the cerebellum are differentially associated with motor activity. Bernard et al. noted a disruption in the connection of the cerebellum with both the striatum and the medial temporal lobe during normal brain aging [224].

## 5. Factors of Accelerated Aging

Factors affecting biological aging fall into two categories: programmed factors and damage-related factors. Programmed aging refers to innate functions that decline or change over time, such as shortened telomeres, reduced growth hormone production, dysregulation of reproductive hormones, and weakened immune responses. Damage-related factors result from normal damage at the cellular level and slowly accumulate to cause aging. These factors usually result in cellular injuries when they outrange the body’s repair capacity. The best examples of damage-related factors include improperly metabolized cellular waste, insufficiently repaired DNA damage, and free radicals derived from normal metabolism or environmental toxins. Both programmed and damage-related aging factors can impair cellular function and increase the brain’s vulnerability to injuries or other harmful stimuli. Recent studies demonstrate that aging can exacerbate the damage and dysfunction of different components of the neurovascular unit (NVU) and thus accelerate the progress of brain injury. The process of energy metabolism causes damage-related aging factors, such as oxidative stress, which may cause injuries to cells. Some injuries are reversible, but some are not. The irreversible injuries accumulate over time and eventually disrupt normal cellular functions. Neurons, with their high metabolic rate, appear to be the most susceptible cell type to the damage-related factors of aging [225].

In general, psychiatric and neurological disorders are frequently linked to an expedited process of brain aging, although the evidence is mixed [107].

Reported evidence demonstrate that prenatal alcohol exposure has profound effects on glial cells [226,227]. In vivo studies indicate that prenatal alcohol exposure interferes with myelinogenesis and is associated with neuroglial heterotopias and abnormal astrogliogenesis. Studies using primary cultures of rat cortical astrocytes show that ethanol affects DNA, RNA and protein synthesis; reduces the number of mitotic cells; alters the content and distribution of several cytoskeletal proteins, including the astroglial marker, glial fibrillary acidic protein (GFAP), and the levels of plasma-membrane glycoproteins; reduces the capacity of astrocytes to secrete growth factors; and induces oxidative stress. Recent evidence suggests that ethanol disrupts the *GFAP* transcription process, resulting in decreased *GFAP* gene expression during astrogliogenesis [226]. It is noteworthy that there are sex-specific differences in the impact of alcohol on brain structures. Morphological characteristics of the brains of men and women afflicted with alcoholism exhibit distinctions compared to those of the same sex [228]. A comparative analysis of the effects of alcohol on cortical thickness and myelinated fiber density in the prefrontal cortex between males and females revealed that men exhibited a more pronounced decrease in cortical thickness and a greater reduction in the density of myelinated fibers in this region, whereas women demonstrated a more significant reduction in neurogenesis in the hippocampus in response to alcohol [229].

Disturbances in the immune system often occur against the backdrop of metabolic changes that are observed with aging [230]. Microglia, a kind of immune system of the central nervous system, perform many important tasks: they protect against pathogens, clean cells, and help maintain and remodel synapses [231,232,233]. These inflammatory responses are protective; with age, microglia become more reactive, increasing the inflammatory response in the brain while reducing the production of beneficial anti-inflammatory molecules [234,235]. Studies in mice show that excessive microglia activity can impair cognitive abilities [236]. Sex-related disparities in brain inflammatory factors, as well as the activation of astroglia and microglia with advancing age, have been documented [106]. Yanguas-Casás et al. [237] used an in-vitro-aged microglia model and microglia isolated from adult (5-month-old) or aged (18-month-old) mice and observed an increase in the phagocytosis of neural debris with aging in both male and female cells. Notably, that the intensity of phagocytosis is higher in aged female microglia than in male ones. Sex-specific differences in microglial population were observed in male and female rats during the early postnatal period, and these disparities endured into adulthood [238]. Cyr and de Rivero Vaccari also found that in female mice, the amounts of cytokines and chemokines expressed in the cerebral cortex increase with age [237,239]. Furthermore, the levels of age-related induction of neuroinflammatory genes in the brain cells of males and females differed: increased expression was observed in females [134,240]. Elderly females had 25–40% more activated microglia, cytokine-secreting resident immune cells in the brain, and astrocytes in the hippocampus compared to males of the same age [241]. Numerous studies have highlighted the correlation between age-related cognitive decline and inflammatory processes [242,243]. In our recent review, we examined the implication of astroglia in neuroinflammation, its role in the aging process and AD pathogenesis, and the general physiological functions of astroglia along with reported sex-related functional disparities in these cells [95].

New evidence suggests that dietary factors play an important role in determining whether the brain successfully ages or develops a neurodegenerative disorder. A higher WHR (waist–hip ratio) may be associated with neurodegenerative, vascular, or metabolic processes that affect brain structures that underlie cognitive impairment and dementia [244]. Research conducted on older individuals has revealed a correlation between obesity and brain atrophy across various brain regions [245] as well as structural modifications in the brain [246,247] and expedited brain aging [248]. Several studies have established a link between higher body mass index (BMI), a gauge for assessing obesity, and accelerated brain aging. Notably, this association has been observed to be more pronounced in men compared to women [249]. Moreover, it is essential to highlight the interplay between cognitive impairment, neurodegenerative diseases, and metabolic disorders with respect to sex. For instance, older women with type 2 diabetes face a 19% greater risk of vascular cognitive impairment than men [250]. Additionally, while elevated total cholesterol levels are significantly associated with AD symptoms in men, this relationship does not hold true for women despite their higher total cholesterol values [251]. Drawing from MRI data involving 623 cognitively normal individuals, Armstrong et al. have identified significant factors influencing age-related changes in brain volume, which are indicative of neurodegeneration. These predictive factors include hypertension, levels of high-density lipoprotein, and the *APOE ε4* carrier status [252].

Diet may influence the development of sporadic or genetic diseases by influencing molecular cascades that either promote or prevent neuronal degeneration. Epidemiological findings suggest that high-calorie diets and folic acid deficiency increase the risk of developing AD and PD. Studies of animal models of these disorders have shown that dietary restriction (reduced calorie intake or intermittent fasting) and supplementation with folic acid can reduce neuronal damage and improve behavioral outcome. Animal studies have shown that the beneficial effects of dietary restriction on the brain result in part from increased production of neurotrophic factors and cytoprotective protein chaperones in neurons. By keeping homocysteine levels low, folic acid may protect brain vessels and prevent the accumulation of DNA damage in neurons caused by oxidative stress and facilitated by homocysteine. A high-calorie diet and elevated homocysteine levels may make the brain vulnerable to age-related neurodegenerative disorders, especially in individuals with a genetic predisposition to such disorders [253].

Summing up, the main factors of accelerated aging of the brain are as follows:

1. Consumption of alcohol, some drugs, and medicines.
2. Injuries, including those received during the neurosurgical intervention.
3. Ischemic changes, atherosclerosis, and chronic anemia.
4. Diet.
5. Overwork and stress.
6. Insomnia.

## 6. Factors of Slow Aging

It has been shown that maintaining an active lifestyle and participating in certain activities can help prevent age-related cognitive decline and dementia. However, some people have a greater ability to resist pathological changes in the brain, such as the accumulation of amyloid protein, due to greater brain reserve. This hypothesis states that higher levels of education, higher socioeconomic status, and basic intelligence protect against the clinical manifestations of brain disease [12].

Many exogenous modulators of neural activity, such as physical activity, enriched environments (e.g., containing tunnels, platforms, toys, and running wheels), calorie restriction and vitamin E, curcumin, resveratrol, blueberry polyphenols, sulforaphanes, salvionic acids, PUFAs (e.g., omega-3 and DHA), LMN diet (La Morella Nuts proprietary diet enriched with polyphenols and PUFAs) regulate and stimulate adult cells—precursors and neurogenesis. In addition, dietary phytochemicals, which are known to have many neurogenic properties, slow brain aging and the progression of neurodegenerative diseases. Although the molecular mechanisms by which these compounds influence neurogenesis have not yet been established. These compounds have been shown to reduce oxidative stress and neuroinflammation, enhance cell signaling, activate autophagy, and influence growth factors [254]. Physical activity and diet modulate common substrates of neuroplasticity (neurotrophic signaling, neurogenesis, inflammation, stress response and antioxidant defense) in the brain, while cognitive activity enhances brain and cognitive reserves. A neuroanatomical study of individuals aged 55 to 79 years found that age-related declines in neuronal density in the frontal, temporal, and parietal cortices and hippocampus were significantly reduced as a function of cardiovascular health; an interesting fact considering that these areas underlie executive function and yet show the greatest rate of aging. Another study of older adults showed a direct correlation between increased levels of PA and improved cognitive function [255].

ALA acid deficiency alters the course of brain development, disrupts the composition of brain cell membranes, neurons, oligodendrocytes, and astrocytes, as well as subcellular particles such as myelin, nerve endings (synaptosomes), and mitochondria. Dietary omega-3 fatty acids are involved in the prevention of some aspects of ischemic cardiovascular disease (including at the level of cerebral vascularization) and some neuropsychiatric disorders—in particular, depression—as well as dementia, including AD and vascular dementia. The effect of omega-3 fatty acids on major depression and bipolar disorder (manic-depressive illness) is under evaluation. Their dietary deficiency (and altered liver metabolism) may inhibit membrane renewal and therefore accelerate brain aging [256].

Anthocyanins from chokeberry fruit increase the structural stability of Klotho (an aging inhibitory protein). Anthocyanins blocked age-related decline in cognitive and responsiveness in accelerated aging mice. In addition, mice treated with anthocyanins showed a better balance of redox systems (SOD, GSH-PX, and MDA) in all age tests. The three major monoamines were norepinephrine, dopamine, and 5-hydroxytryptamine, and their levels were significantly elevated; transcription levels of inflammatory cytokines (COX2, TGF-β1 and IL-1) and DNA damage were significantly reduced in the brains of anthocyanin-fed mice [257].

It is also important to note the reported effect of bilingualism on delaying cognitive age-related changes in a number of studies [258,259,260]. Notably, a delay in the onset of clinical symptoms of AD has been reported in bilinguals compared to monolinguals [261]. In addition, it has been reported that lifelong music practice also prevents age-related cognitive decline and improves brain plasticity [262,263].

### 6.1. Estrogenic Support for Neuronal Vitality

Estrogens are a group of pleiotropic steroid hormones with diverse mechanisms of action in various physiological systems. Estrogens perform such “non-reproductive” functions as ensuring the development and differentiation of brain cells at various stages of ontogenesis, influencing the neuroendocrine regulation of metabolic processes and regenerative and plastic processes in the central nervous system (CNS), ensuring the formation of behavioral, psychological and sexual reactions, training, memory, anxiety and mood [264,265,266,267]. Estrogens have an important protective effect on the CNS: they slow down the processes of apoptosis of CNS cells and increase their survival under hypoxia, hypoglycemia, and intoxication conditions [268]. Estrogen receptors have been found in the hypothalamus, pituitary, hippocampus, and frontal cortex, indicating that estrogen plays a role in brain development. Gonadal hormone receptors have also been found in the basal forebrain nuclei [269]. Sex steroids are involved in the formation of cognitive functions; they reduce the clinical manifestations of depression and other mental disorders [270,271,272]. Estrogen receptors alpha (ERα) and beta (ERβ) belong to the so-called classical estrogen receptor family, while G-protein-coupled estrogen receptor 1 (GPER-1) is a non-classical estrogen receptor located primarily in the plasma membrane. Estrogens can stimulate mitochondrial biogenesis [273], acting as antioxidants, promoting DNA repair, inducing the expression of growth factors, and modulating cerebral blood flow. In addition, estrogen-dependent signaling pathways are involved in regulating the balance between the proliferation and differentiation of neural stem/progenitor cells (NSPCs), thereby influencing neurogenic processes [274]. Studies show that lower estrogen levels as a result of oophorectomy and hysterectomy have a negative effect on brain volume. Less pronounced brain aging was reported in females with a history of multiple births, highlighting a potential link between sex hormone exposure and brain aging [275]. Estrogen use in senior females was associated with the preservation of gray and white matter volumes in areas of the brain associated with cognitive function, including the frontal, temporal, and parietal lobes. These results remained statistically significant regardless of history of estrogen use time, cardiovascular risk factors, genetic make-up, or lifestyle factors [276,277] and obesity [278], and animal studies indicate that estradiol is the most important estrogen for maintaining hippocampal function [122,279,280]. The gonadal steroid estrogen can potentially affect the viability of basal forebrain cholinergic neurons [281]. It has been noted that headaches—in particular, migraines—are more common in women than in men, especially during the reproductive period [282].

Several in vitro and in vivo studies show that estradiol and metabolites derived from phytoestrogens such as trans-resveratrol promote the survival of neurons exposed to a variety of stressful conditions [283,284]. Some conjugated equine estrogens (such as premarin) are widely used to reduce menopausal symptoms, particularly by increasing functional neuronal activity, counteracting the cognitive decline associated with aging and AD [285,286,287,288,289]. The activating effect of these hormones on glial proliferation plays a dual role: on the one hand, gliosis is necessary for the restoration of neuronal tissue; on the other hand, it can contribute to the replacement of neurons by stromal elements [290].

#### 6.1.1. Antioxidant Activity

Various neurodegenerative diseases are characterized by a decrease in mitochondrial activity, a decrease in oxidative phosphorylation, and an increase in the production of reactive oxygen species (ROS) in the CNS [291]. Oxidative stress is also increased due to a loss in antioxidant properties and an increase in production of inflammatory cytokines; both processes gradually expand with age. Mitochondria are considered the main ROS producer [292]. Estrogens can activate the production of reactive oxygen species by increasing the activity of mitochondria and the redox cycle of estrogen metabolites [293]. On the other hand, the phenolic hydroxyl group of estrogens can act as an antioxidant agent, being a protective factor against cardiovascular and neurodegenerative diseases [293].

#### 6.1.2. DNA Repair

Oxidative modification of bases can lead to transcriptional mutagenesis [294] or DNA single-strand breaks (SSB) [295], two processes that lead to genomic instability and cell death [296]. Base excision repair (BER) is one of the major factors contributing to repair. Its impairment is associated with brain aging and age-related neurodegenerative diseases [23]. Estrogens activate the transcription of *APE1* and *NTH1* [297], contributing to maintaining a high BER level. It was also shown that estrogens enhance the transcription of DNA repair enzymes in the cerebral cortex after hypoxia, helping to reduce oxidative stress [274].

#### 6.1.3. Growth Factors

The neuroprotective functions of estrogens are closely related to the synthesis of growth factors. Brain-derived neurotrophic factor (BDNF) is a neurotrophin essential for brain development, neurogenesis [298,299], neuronal survival, synaptic plasticity, and memory formation [300,301]. Estrogens can induce *BDNF* expression by direct binding to the estrogen response element (ERE) in the *BDNF* gene [298]. Furthermore, it has been demonstrated that BDNF expression in certain brain regions may be influenced by sex. Specifically, sex-specific BDNF expression has been observed in the mossy fiber pathways within the hippocampus of rat brains. Notably, estradiol has been linked to elevated levels of BDNF expression, whereas testosterone has been found to inhibit it [302]. Intriguingly, BDNF expression levels decline with age, and this decrease has been linked to cognitive decline in elderly females but not in males [303].

#### 6.1.4. Synaptic Plasticity

Estrogens also play a neuroprotective role by promoting synapse formation. Interestingly, estrogen receptors ERα and ERβ are located in many regions of the brain, with subcellular localization in synaptic terminals and dendritic spines, dendritic trunks, axons, mitochondria, and glial cell processes [304], which indicates a role in local regulation of synaptic transmission [23]. Estrogens are essential for axonal growth and synaptic plasticity [305,306], improving performance in various memory and cognitive tests [307].

#### 6.1.5. Neurodegenerative Disorders

Experimental and clinical evidence suggests that estrogens have a protective effect against neurodegenerative diseases [289,293]. Females who experience premature menopause before age 40 and have not received estrogen treatment show an increased risk of developing cardiovascular and neurodegenerative disorders, which correlate with increased mortality [308]. Hormone replacement therapy has been shown to be highly effective in the development of AD and PD in postmenopausal women, although postmenopausal women have a higher prevalence of this pathology than men [142,309,310] and more rapid cognitive decline, suggesting a possible role of sex hormones in the development of AD. AD is characterized by a progressive decrease in the number and maturation of adult neurons in the dentate gyrus of the hippocampus [311], indicating an impairment of neurogenesis in the hippocampus that is closely associated with loss of hippocampal function and cognitive decline [312,313]. Estrogen treatment increases the number of proliferating cells and synaptic contacts in the hippocampus [314] with increased proliferation of neuronal precursors in the dentate gyrus.

Clinical evidence suggests that women with low estrogen levels may develop PD earlier than women with high estrogen levels [315,316,317,318,319]. Estrogens can positively influence dopamine neurotransmission by decreasing the expression of catechol-O-methyltransferase (COMT), an enzyme responsible for the degradation of dopamine [320].

In addition, the protective effects of estrogens in PD are also associated with activation of the mitogen-activated protein kinase (MAPK) signaling pathway and subsequent activation of Bcl-2, as shown in dopaminergic cells in mesencephalic slice cultures, as well as activation of BDNF and effects on oxidative homeostasis [319,321,322]. Estrogens can also act as anti-inflammatory agents in the wall of blood vessels, protecting it from cytokines and free radicals during ischemia of various origins [323].

In conclusion, one could say that the neuroprotective role of estrogens implies a close regulation of such processes as antioxidant activity [293], DNA repair [297], growth factor synthesis [324], synaptic plasticity [304], and improved microcirculation [325]. These mechanisms are vital in maintaining neuronal survival and countering the increased risk of age-related cognitive decline and neurodegenerative disorders [285,326]. Estrogen promotes cognitive function by affecting areas of the brain such as the prefrontal cortex and the hippocampus [304]. Local synthesis of estrogens occurs in the hippocampus of both sexes. Hippocampal estrogen modulates memory-related synaptic plasticity. The slow action of 17β-estradiol (17β-E2) occurs via classical nuclear receptors (ERα or ERβ), while the fast action of E2 occurs via synapse-localized ERα or ERβ. An increase or decrease in E2 concentration rapidly changes the density and morphology of spines in neurons CA1-CA3. ERα, but not ERβ, stimulates spinogenesis [327]. Xenoestrogens, commonly found in plastic polycarbonate polymers such as mineral water bottles and food can lacquers, can act as endocrine disruptors that significantly alter estrogen-mediated signaling pathways [328,329].

## 7. Conclusions

**Figure 6:**
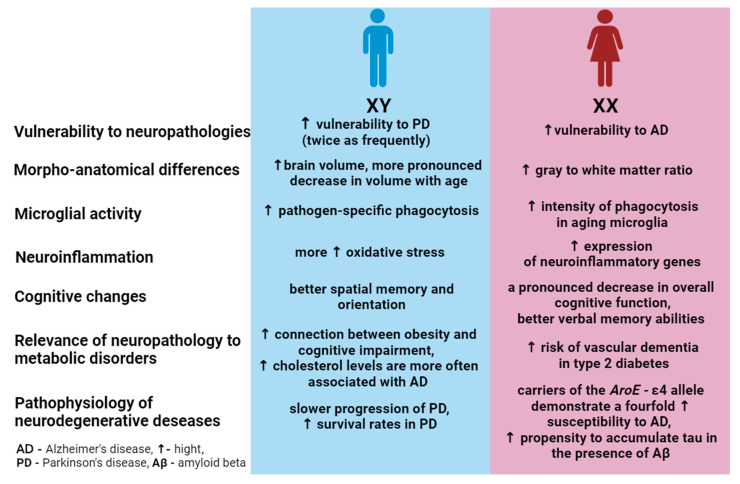
Reported sex differences in age-related brain processes.

Certainly, the process of brain aging is intricate and multifaceted, encompassing an array of interlinked irreversible processes occurring across tissue, cellular, and molecular levels. In addition to well-known external and genetic factors contributing to the risk of age-related neurodegenerative conditions and fostering accelerated aging, the presence of sexual dimorphism in these processes adds a significant layer of interest. Evidence from cognitive, neuroanatomical, and neurophysiological studies frequently underscores the pivotal role of sex differences in modulating aging dynamics and functional alterations in the brain. Anatomical disparities stemming from varying rates of neurodegeneration in distinct brain regions across sexes underscore the escalating age-associated divergence in cognitive abilities, notably the preservation of verbal memory in women and enhanced learning and spatial orientation in aging men. Sex-related differences are noted in epidemiology, pathophysiology, clinical manifestations, disease progression, and response to treatment (Figure 6). Features of immunological reactions, the level of sex hormones, and the activity of free radical processes, which exhibit considerable divergence between males and females, can also serve as the basis for the development of new methods for the metabolic correction of age-related neurodegenerative reactions.

The role of sex hormones, particularly estrogens, in neuroprotection is particularly captivating. A central factor in the distinct incidence of conditions like AD and PD among older men and women is the influence of estrogens and their robust neuroprotective function. In spite of the multitude of exogenous and endogenous factors hastening age-related degeneration, the prospect of neuroprotection of brain cells in the age aspect remains of fundamental importance. Along with estrogens, the activity of the antioxidant system, DNA repair mechanisms, and the balance between pro-inflammatory and anti-inflammatory responses hold paramount significance. While these factors possess a universal nature when considering geroprotective mechanisms across the entire body, they take on unique characteristics due to the absence of fundamental antioxidant enzymes in the brain, the brain’s immune privilege, and the incapacity of differentiated neurons to divide. These distinctive features of the exceedingly intricate organ that underscores our individuality, combined with pronounced sex differences, make the exploration of brain aging a particularly captivating field of study. This domain presents opportunities for an individual approach to correcting pathological processes associated with age.

## Data Availability

The raw data supporting the conclusions of this article will be made available by the authors without undue reservation.
